# Application of an Activated Carbon-Based Support for Magnetic Solid Phase Extraction Followed by Spectrophotometric Determination of Tartrazine in Commercial Beverages

**DOI:** 10.1155/2015/291827

**Published:** 2015-03-19

**Authors:** José A. Rodríguez, Karen A. Escamilla-Lara, Alfredo Guevara-Lara, Jose M. Miranda, Ma. Elena Páez-Hernández

**Affiliations:** ^1^Laboratorio 2, Área Académica de Química, Universidad Autónoma del Estado de Hidalgo, Carretera Pachuca-Tulancingo Km. 4.5, 42184 Mineral de la Reforma, HGO, Mexico; ^2^Departamento de Química Analítica, Nutrición y Bromatología, Facultad de Veterinaria, Universidad de Santiago de Compostela, Pabellón 4 Planta Baja, Campus Universitario, s/n, 27002 Lugo, Spain

## Abstract

A method is presented for magnetic solid phase extraction of tartrazine from nonalcoholic beverages. The method involves the extraction and clean-up by activated carbon covered with magnetite dispersed in the sample, followed by the magnetic isolation and desorption of the analyte by basified methanol. The tartrazine eluted from the magnetic support was determined by spectrophotometry. Under optimal conditions, the linear range of the calibration curve ranges from 3 to 30 mg L^−1^, with a limit of detection of 1 mg L^−1^. The method was validated by comparing the results with those obtained by HPLC. A precision of <5.0% was obtained in all cases and no significant differences were observed (*P* < 0.05).

## 1. Introduction

Dyes are a group of additives used to improve the appearance of foods, textiles, and medicine allowing the homogenizing and the assertion of the color, which is the most important sensory characteristic [1]. Synthetic dyes have been used because they are more resistant to changes in temperature and acidity [2] than natural dyes. All of these compounds contain a chromophore group and different substituents resulting in a large group of synthetic dyes, each with particular chemical properties.

The azo dyes are the most commercially used group of synthetic dyes. The main feature of these compounds is the presence of an azo group (–N=N–) and aromatic groups, resulting in a substance with high electron conjugation and a high molar absorption coefficient, making it useful at low concentrations [3].

Tartrazine, also known as yellow 5 or E 102 dye, is one of the most important synthetic azo dyes used in the food industry to confer a yellow color to food. It is soluble in water (14 mg/100 mL) giving a yellow color to the solution. It is usually applied in pastries, baked goods, snacks, drinks, biscuits, ice cream, and other sweets [4].

In spite of its useful qualities, tartrazine has also been associated with allergenic effects, hyperactivity, and attention deficit disorder [5]. For this reason, the toxicity of tartrazine was studied in the early 60s by the Expert Committee on Food Additives of FAO/WHO, which established an acceptable daily intake (ADI) of 7.5 mg kg^−1^ of body weight per day. Moreover, since 2010, foods containing tartrazine must carry the warning “may alter the activity and attention in children.” In Mexico, this dye is regulated according to the Official Mexican Standard NOM-218-SSA1-2011 which allows 100 mg L^−1^ as the maximum amount of tartrazine in commercial beverages [6].

In general, the methodology for the quantification of dyes involves a sample treatment, identification, and quantification steps. The chosen method depends on the type of food and the lipid, protein, and carbohydrate content. Other properties such as acid-base properties of the analyte and the interferences present in the sample matrix are also important [7]. Thus, when liquid chromatography, capillary electrophoresis, and electrochemical techniques are used, it requires a simple treatment of the sample, usually dilution and subsequent filtration. However, these instrumental methods have certain disadvantages such as the high cost of analysis (equipment and reagents) and lower analysis rate (2–4 samples per hour) [8, 9]. On the other hand, if the determination of dyes is completed using a quick and simple spectrophotometric technique, it must be considered that the presence of preservatives (sodium benzoate or citrate) and proteins may interfere significantly during the quantification step. According to this, the isolation of the sample dye using different extraction materials is critical during the analytical process [9].

Different separation strategies have been evaluated for dye extraction. Anion exchange, where acid dyes are retained by sulfonic groups present in the polymer structure [10], and liquid-liquid extraction (*n*-butanol-water) based on the formation of ion pairs with trimethyloctadecylammonium salts [11] have been proposed. Solid phase extraction is another technique used to isolate dye from complex matrices. The solid phases used for this purpose were zeolite or silica C18; the retained dye was eluted from the solid phase with methanol [12, 13].

Additionally, activated carbon has been proposed as colorant adsorbent because it has an excellent surface area and a well-defined pore structure that favors the retention of the analyte [14–17]. Despite its versatility, the activated carbon has the disadvantage of being difficult to separate from the aqueous matrix where it was dispersed. In order to facilitate separation of the extraction support from the liquid phase, Šafaříková and Šafařík [18] propose the magnetic solid phase extraction (MSPE). MSPE is based on the use of magnetite-modified activated carbon as the solid phase with paramagnetic properties. For this reason, the support can be easily isolated from the dispersion medium by applying an external magnetic field. The main advantages of MSPE are as follows: (1) presenting the possibility of using large volumes of samples, greatly reducing the time of pretreatment and the analysis result; (2) having great interaction between analytes and the solid phase with the full dispersion of the adsorbent in the sample; and (3) providing easy isolation of the adsorbent from the analytical matrix, reducing the risk of loss of analyte [19].

In spite of their promising qualities, the MSPE has been used mainly for the separation of antibiotics and other organic molecules [20]. The use for the extraction of dyes has been limited to the determination of Reactive Red 198 [21], and methylene Blue [22]. In all cases, the main advantage of the adsorbent support was its selectivity in the extraction of the analyte and the possibility of using volumes from 100 to 1000 mL [18], providing shorter analysis times as compared to conventional techniques.

According to the above-mentioned, the purpose of this work is to design a methodology based on magnetic solid phase extraction using magnetite-modified carbon for the spectrophotometric analysis of tartrazine. The method was used for the analysis of this dye in a complex matrix of nonalcoholic beverages.

## 2. Materials and Methods

### 2.1. Synthesis and Characterization of Magnetite-Modified Carbon

For the synthesis of the magnetite-modified carbon, a commercial activated vegetable carbon from Clarimex S.A. de C.V. was used. The synthesis was performed in two stages: in the first stage, magnetite was obtained by precipitation and its partial oxidation of iron (II) sulfate heptahydrate FeSO_4_·7H_2_O (3.6 g). The iron precursor was dissolved in 100 mL of deionized water, stirring constantly and keeping at 60°C. The pH solution was adjusted to 10.0 ± 0.2 and a stream of air was passed through the reaction mixture. The initial green precipitate (Fe(OH)_2_·*x*H_2_O) turned black after 40 minutes of reaction as a consequence of the partial oxidation of Fe(II) to Fe(III) by the action of the O_2_ from the air stream; the black color of the precipitate is characteristic for Fe_3_O_4_ [23]. In the second step, 1.0 g of activated carbon was added to the reaction vessel and the mixture was stirred for 30 minutes. The magnetic phase was separated with a magnet and washed three times with distilled water. The solid phase was dried at 60°C for 24 h. The solid phase was pulverized in an agate mortar and stored in a desiccator until use.

In order to carry out the characterization of the synthesized magnetite-modified carbon, various instrumental techniques were used. X-ray powder diffraction analysis was performed in a Philips PW1710 instrument equipped with a copper anode and an automatic divergent opening. The conditions for the analysis were 1.54 Å CuK*α* radiation; 40 kV voltage tube; 30 mA current tube; 0.500 intensity ratio (*a*2/*a*1); 1° divergence slit; 0.1 receiving slit; (2*θ* °) 5 initial angle; (2*θ* °) 70 end angle.

Morphological analysis of the solid was performed in a scanning electron microscopy (SEM) JEOL JSM-820. Qualitative analysis and determination of the distribution of magnetite in the solid were performed with a LINK QX-2000 analyzer by Energy Dispersive X-ray Spectroscopy. All spectra were obtained at 15 kV, a distance of 39 mm, and 2,500 counts; the detector angle relative to the sample in all cases was 45°.

### 2.2. Tartrazine Extraction-Elution

For the extraction studies, 0.05 g of magnetite-modified carbon was mixed with 20.0 mL of aqueous solution of tartrazine and stirred mechanically for 30 minutes. The pH value for tartrazine solution was varied using acetate (pH = 5.0), phosphate (pH = 7.0), and borate (pH = 9.0) solutions at 1.0 mol L^−1^ concentration. After the extraction, the solid phase was separated using a neodymium magnet and the remaining liquid phase was analyzed by spectrophotometry at a wavelength of 434 nm in a UV-Vis spectrophotometer HACH DR-2700 with a quartz cell with 1.0 cm of path length. Thus, the remaining tartrazine was quantified by interpolation in a calibration line constructed with standard solutions of tartrazine (10.0–100.0 mg L^−1^) prepared in the respective buffer solution. A control experiment was carried out using activated carbon in order to evaluate the effect of the magnetic modifications in support of the tartrazine extraction.

For the elution of the retained tartrazine from the synthetized support, several eluting systems were evaluated following this procedure in triplicate: 50 mg of solid phase containing tartrazine was mixed with 2.0 mL of eluent and stirred using ultrasound for 5 minutes. 2 mL of the resulting solution was transferred to a 10 mL volumetric flask and filled with eluent solution up to the mark. The eluted tartrazine was quantified by interpolation in a calibration line constructed with absorbance values of standard solutions of tartrazine (10.0–100.0 mg L^−1^) prepared in the respective eluent solution.

### 2.3. Analysis of Real Samples

Six samples of commercial beverages containing tartrazine were analyzed in triplicate following this protocol: 10 mL aliquot of the drink was mixed with 2.5 mL of 1.0 mol L^−1^ acetate buffer solution and transferred to a 25 mL volumetric flask and then deionized water was added up to the mark. Later, 20.0 mL of this solution was placed in a polypropylene tube containing 75 mg of magnetic support and mechanically stirred for 30 minutes in order to extract the dye. After the extraction, the solid phase was removed from the aqueous matrix using a neodymium magnet. The solid phase was then mixed with 1.0 mL of eluent and stirred for 5 minutes using ultrasound to remove the extracted tartrazine. The liquid phase was transferred to a 10 mL volumetric flask and filled with elution solution up to the mark. The concentration of tartrazine in the last solution was determined spectrophotometrically.

In order to evaluate the proposed methodology, the amount of tartrazine in the commercial beverages was also analyzed using an HPLC. This method considers a calibration line using standard solutions of tartrazine from 5 to 20 mg L^−1^. Samples were prepared as follows. An aliquot of 0.5 mL was diluted adding a mobile phase up to 5 mL. In order to introduce the samples to the chromatographic system, it was necessary to filter it using membranes of 0.45 *μ*m pore size.

## 3. Results and Discussion

### 3.1. Characterization of Modified Carbon

**Figure 1:**
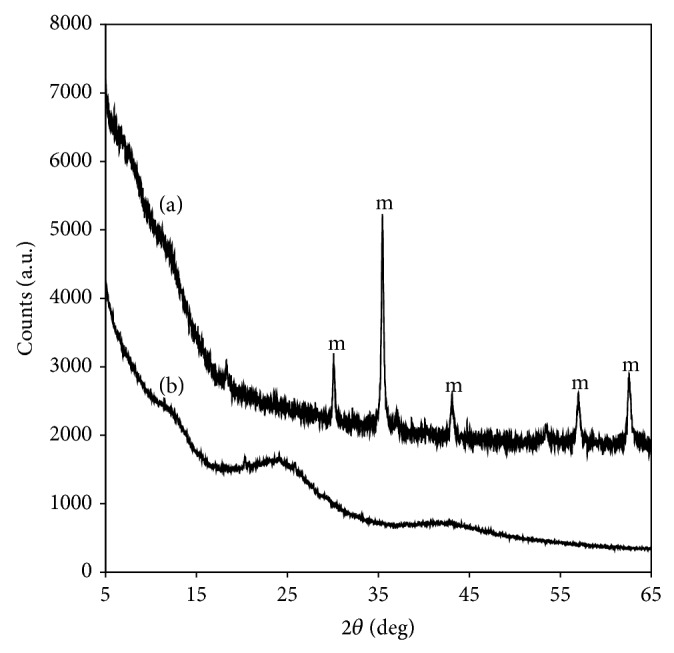
Diffractogram of (a) magnetic modified carbon support and (b) activated carbon.

Instrumental techniques were used to provide information about the composition and structure of the magnetic modified carbon. Thus, the X-ray diffraction (XRD) studies were performed in order to determine the iron oxide form present in the solid. The XRD diffractograms from Figure 1(a) show the signals labeled as “m” that correspond to the characteristic diffraction lines of Fe_3_O_4_ (2*θ* = 30.1°, 35.5°, 43.1°, 53.4°, 57.0°, and 62.6°) according to the Joint Committee on Powder Diffraction Standards [24]. The broadband signal observed between 20° and 30° for the 2*θ* angle (Figure 1(b)) is characteristic of amorphous carbon, the raw material.

**Figure 2:**
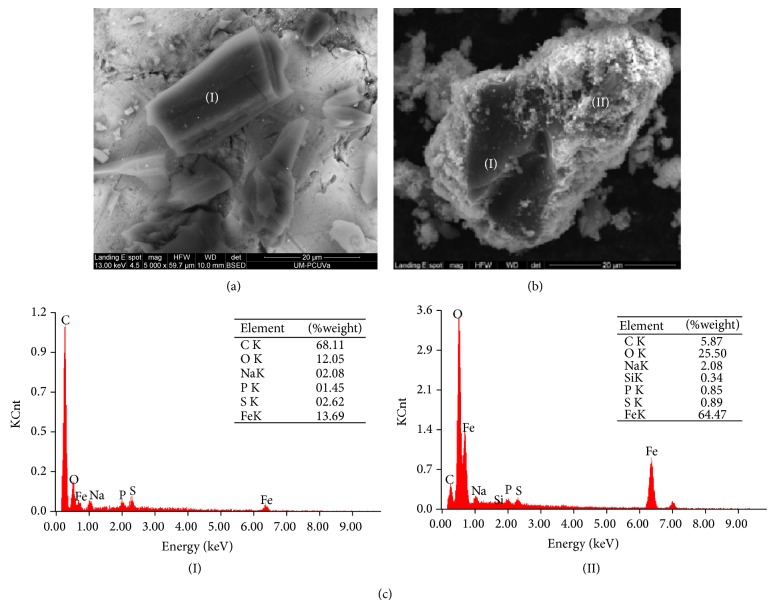
(a) Micrograph for activated carbon; (b) micrograph for magnetic modified carbon support; (c) energy dispersive spectra obtained from the analysis of zones I and II of magnetic modified carbon support.

The morphological study with scanning electron microscopy (SEM) for activated carbon shows a uniform phase with inhomogeneous particle sizes greater than 20 *μ*m (Figure 2(a)). The micrograph is consistent with SEM studies performed without modifying the activated carbon [25]. In the case of the synthetized support, it can be seen that the activated carbon is covered by a phase with a smaller size (Figure 2(b)). This was confirmed with the energy dispersive spectrum (Figure 2(c)) that shows a larger amount of carbon for the section (I), while the Fe-content is greater for the area (II), so it is concluded that the magnetite phase is covering the activated carbon.

### 3.2. Study of Chemical Variables for the Extraction of Tartrazine with the Magnetic Modified Carbon Support

**Figure 3:**
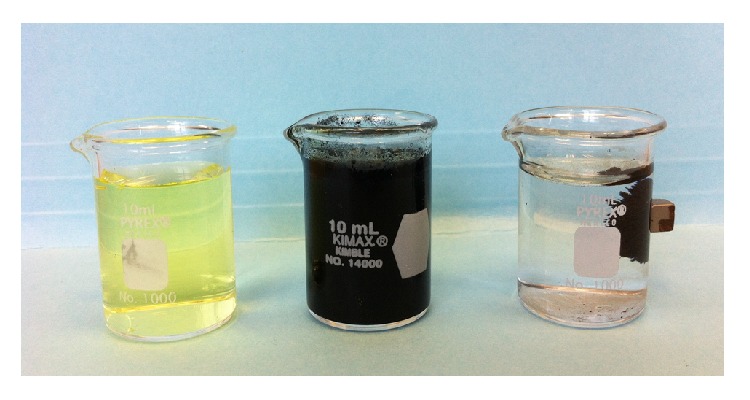
Adsorption experiment. From left to right: dissolution of tartrazine, mixture of tartrazine with magnetic modified carbon, and magnetic support separation.

The extraction of tartrazine from standard solutions using the synthetized modified carbon is shown in Figure 3. It is possible to visually verify the cleaning process of the tartrazine solution.

**Figure 4:**
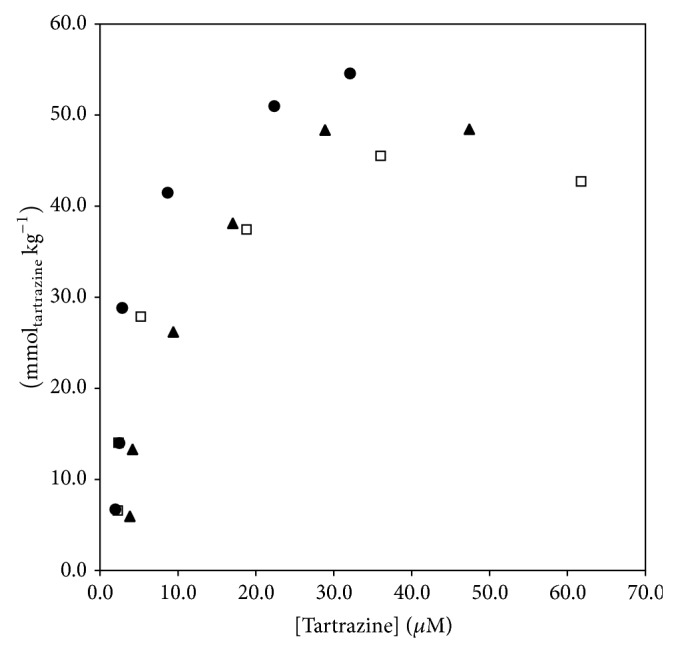
Adsorption isotherms for tartrazine on magnetic carbon modified at pH values (∙) 5.0, (▲) 7.0, and (□) 9.0.

The capability of the synthetized modified carbon for the extraction of tartrazine was also evaluated at different pH values. The results shown in Figure 4 correspond to acidic, basic, and neutral medium adsorption isotherms constructed by plotting the concentration of tartrazine in the solution at equilibrium (*μ*M) against the concentration of the sorbate on the solid phase (mmol kg^−1^) after adsorption. Additionally, Table 1 shows the maximum quantity of adsorbate on the solid support at different pH values.

According to Figure 4 and Table 1, the decrease of pH values results in the increase of the amount of absorbed tartrazine. This is consequence of the interaction of tartrazine in its anionic form with the acid form of the magnetite at low pH values. In basic medium a repulsion between the negative charges of tartrazine in solution and the support is reflected in the reduction of the adsorbate retention [26]. Based on these results, pH of 5.0 was selected as the most suitable value for the retention of dye.

As part of the characterization of the MSPE-dye system, the affinity constant value between substrate and tartrazine was estimated. The analysis of the isotherm values using a Scatchard plot demonstrates the value of the affinity constant *K*_*d*_ for the following dissociation reaction [27]:

(1) $$\begin{array}{c} \text{TS}⇆\text{T}+\text{S}, \end{array}$$

where TS corresponds to tartrazine adsorbed on the support, T is the tartrazine in solution, and S is the magnetic modified carbon support.

log⁡⁡*K*_*d*_ values are shown in Table 2, observing a linear trend which is associated with the homogeneity of the support. Because this is a dissociation constant, it follows that the support with a higher affinity is the activated carbon; however, the synthetized support has a suitable log⁡⁡*K*_*d*_ value because in the retention-elution methodologies design, it is recommended that the support has an average affinity to the substrate with log⁡⁡*K*_*d*_ values between −7.0 and −4.0 [20].

Additional studies on the selection of the best chemical conditions for the tartrazine elution adsorbed on the magnetic modified carbon were performed. Thus, several eluting systems were evaluated for the elution step: methanol, acetonitrile, basified methanol, and basified acetonitrile [28, 29]. After the spectrophotometric analysis of the eluted tartrazine, it can be seen that basified methanol provides the greater signal (Table 3) as a consequence of removing a greater amount of tartrazine. For this reason, this eluent was chosen for elution of tartrazine in the following experiments.

### 3.3. Optimization of the Physical Variables of the MSPE Process

To optimize the conditions for the retention and elution of tartrazine involved in the MSPE system, a Taguchi experimental design was performed. The main advantage of this technique is that it provides useful information with minimal experimentation using matrices of special design (orthogonal arrays), in which columns (factors or controllable parameters) and rows (experiments) are accommodated in such a way that a combination of factors and levels of each experiment are indicated [30].

The selected response factor was the percentage of recovery for the analysis of 20 mL of a solution of 30 mg L^−1^ tartrazine. The selected control factors (parameters), each at 3 levels, were sample volume, volume of eluent, mass of magnetic modified carbon support, and NaOH concentration in the eluent [20]. Based on an L9 (34) Taguchi orthogonal array, the matrix design and results obtained are shown in Table 4.

**Figure 5:**
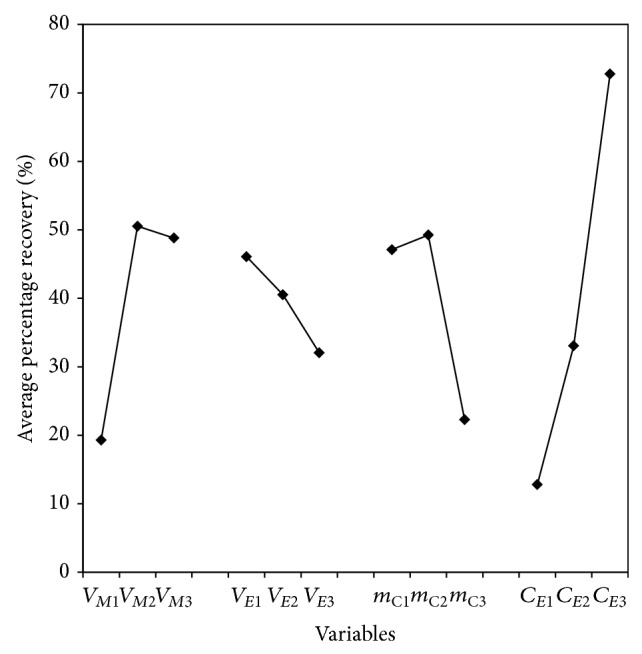
Plot of average effects in the tartrazine recovery. *V*_*M*_: sample volume; *V*_*E*_: eluent volume; *m*_C_: magnetic modified carbon; and *C*_*E*_: NaOH concentration in the eluent.

Figure 5 shows a typical graph obtained by plotting the average percentage of recovery for each factor against each of its levels. According to this, the most suitable conditions for tartrazine adsorption-elution were 20 mL sample, 1 mL of eluent, 75 mg of magnetic modified carbon, and a concentration of 0.25 mol L^−1^ NaOH for the basified methanol solution. These conditions correspond to experiment number 4, which has the highest percentage of dye recovery.

Additionally, the contribution percentage of each variable was determined by ascertaining that NaOH concentration in the eluting solution has the greater effect (45.4%), followed by the sample volume (23.6%), the mass of adsorbent (20.4%), and the eluent volume (10.6%). The higher contribution of the eluting solution in the tartrazine elution step confirms the theory of charge-repulsion between support and analyte, which is favored at a higher concentration of NaOH.

### 3.4. Analytical Parameters of the MSPE Developed Method

Under the optimized conditions described above, calibration lines using tartrazine standard solutions in the concentration range of 5.0 to 30.0 mg L^−1^ were carried out. The obtained signal (AU) was measured in triplicate and the calibration lines were plotted using the average signal of the eluted tartrazine. Calibration lines show a linear dependence between the average signal and the concentration of tartrazine present in the initial standard solution.  Table 5 shows the calibration line regression parameters.

From the values reported in Table 5, it can be seen that the proposed methodology allows for the quantification of tartrazine in drinks at levels established by the Official Mexican Standard NOM-218-SSA1-2011 which allows 100 mg L^−1^ as the maximum amount of tartrazine in commercial beverages [5].

### 3.5. Interference Study

Several nonalcoholic beverages containing tartrazine also have additives as proteins and preservatives that improve physical appearance and shelf life. For this reason, the tartrazine adsorption-elution method was evaluated adding several interfering compounds. The evaluated interferents included casein, egg albumin, acesulfame K, sodium benzoate, aspartame, sodium citrate, glucose, and sucrose. Solutions of each interfering compound were prepared dissolving 10 mg in 10 mL acetate buffer.

To perform the test, 75 mg of magnetic modified carbon was mixed with 20 mL of 30 mg L^−1^ tartrazine solution and 3 mL of interferent solution. After this absorption step, 1 mL of basified methanol with NaOH 0.25 mol L^−1^ was used to elute tartrazine. Then 0.5 mL of the eluted solution was transferred to a 5 mL volumetric flask and filled up to the mark. This solution was analyzed by UV-Vis spectrophotometry. Results did not show a %RSD value higher than 5% of the analytical signal in a similar experiment without interferents. According to this, the proposed compounds do not interfere with the tartrazine determination following the proposed methodology.

### 3.6. Analysis of Real Samples

The tartrazine concentration in six commercially available beverages was determined. The main components of every beverage that were reported by the manufacturer are listed in Table 6.

**Figure 6:**
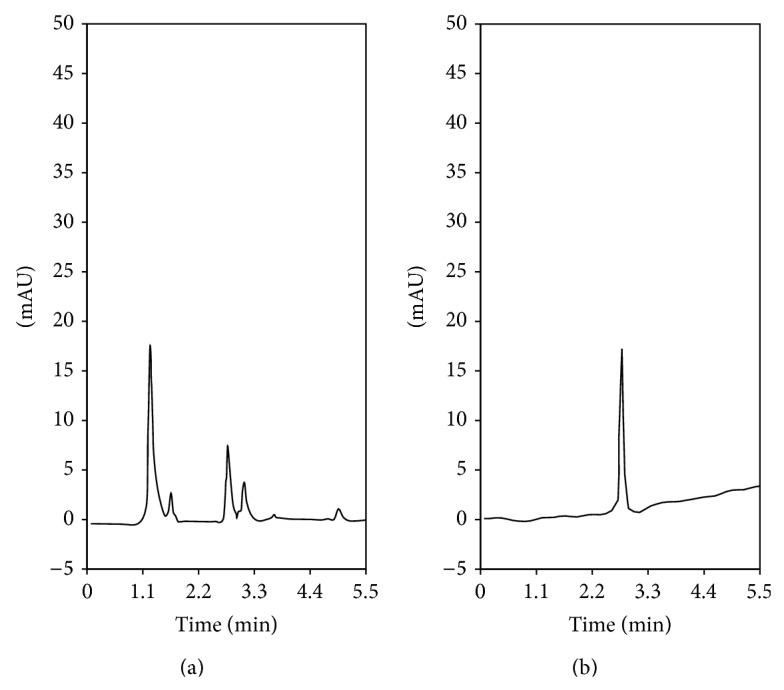
Chromatograms of (a) original sample and (b) eluted tartrazine solution after using MSPE developed method.

Following the developed and optimized method, the tartrazine concentration for each beverage is shown in Table 7. This value represents the average of three independent determinations. Additionally, in Table 7 it is possible to observe the results for the analysis of samples using the HPLC reference method and MSPE-HPLC (Figure 6). For each beverage, the average concentration of tartrazine obtained using both methods was compared using a *t*-test with 2 degrees of freedom and 95% of confidence (*t*_tab_ = 4.3). This analysis revealed no significant differences between the results from each method. Therefore, the methodology of MSPE is comparable with the reference methodology. Additionally, the MSPE is a robust preconcentration technique that can be coupled even to spectrophotometry or HPLC.

## 4. Conclusions

In the present work, an activated carbon covered with magnetite support was synthesized; this support has magnetic properties that allow its separation by applying an external magnetic field.

The best conditions for the extraction and elution of tartrazine were an initial sample volume of 20 mL, buffered with acetate buffer solution, at pH 5, and mixed with 75 mg of magnetic modified carbon, tartrazine elution with 1 mL of NaOH 0.25 mol L^−1^ in methanol.

Thus, the proposed sample treatment coupled to spectrophotometric analysis is an alternative to the analysis of azo dyes in the food industry because the parameters, analytical precision, and accuracy are similar to HPLC methodologies. However, the proposed methodology saves time and is less expensive than the reference method.

## Figures and Tables

**Table 1:** Maximum adsorbed tartrazine on the adsorbent at different pH values.

pH value	Tartrazine adsorbed (mmol kg^−1^)
5.0	54.5
7.0	48.4
9.0	42.7

**Table 2:** Tartrazine magnetic-modified carbon support dissociation constants.

Support	log⁡K_d_
Activated carbon	−28
Magnetic-modified carbon	−6.5

**Table 3:** Results of the evaluation of different systems for tartrazine elution.

Eluent	Signal 1	Signal 2	Signal 3
Methanol	0.6000	0.6010	0.6020
Basified methanol	0.7720	0.7700	0.7700
Acetonitrile	0.1460	0.1460	0.1450
Basified acetonitrile	0.4440	0.4420	0.4440

**Table 4:** Matrix obtained during optimization design for the MSPE system.

Experiment	*V*_*M*_ (mL)	*V*_*E*_ (mL)	*m*_C_ (mg)	*C*_*E*_ (M)	% recovery
1	10.0	1.0	50	0.0025	6.66
2	10.0	2.0	75	0.025	23.48
3	10.0	3.0	100	0.25	27.74
4	20.0	1.0	75	0.25	99.95
5	20.0	2.0	100	0.0025	7.49
6	20.0	3.0	50	0.025	44.10
7	30.0	1.0	100	0.025	31.59
8	30.0	2.0	50	0.25	90.58
9	30.0	3.0	75	0.0025	24.19

**Table 5:** Parameters of the regression line for signal (AU) versus tartrazine concentration (mg L^−1^) plot.

Parameter	Value
Root mean square deviation, *s*_*e*_	0.013
Intercept, *b*_0 _ ± ts (*b*_0_)	0.010 ± 0.031
Slope, *b*_1_ ± ts (*b*_1_)	0.041 ± 0.003
Linear interval (mg L^−1^)	3.0–30.0
Limit of detection (mg L^−1^)	1.0
Repeatability (%RSD, *n* = 3, 10.0 mg L^−1^)	1.8
Reproducibility (%RSD, *n* = 9, 10.0 mg L^−1^)	3.2

**Table 6:** Chemical composition of the analyzed commercial beverages.

Sample	Composition
1	Soy (water and selected soy seeds), sugar, maltodextrin, concentrated pineapple juice, flavor identical to natural, pectin, and tartrazine.
2	Water, sugar, concentrated orange, tartrazine, and sodium.
3	Water, corn syrup, high fructose, concentrated pineapple juice reconstituted, citric acid, pectin, artificial flavor, acesulfame K, and yellow 5.
4	Water, high fructose corn syrup, sugar, citric acid, sodium chloride, sodium citrate, monosodium phosphate, Arabic gum, ester gum, natural lemon-lime flavor, and tartrazine.
5	Water, high fructose corn syrup, concentrated juice (7% orange and 3% pineapple), citric acid, ascorbic acid, acacia gum, ester gum, natural and artificial flavors, sodium citrate, yellow 5 (tartrazine), and beta carotene dyes.

**Table 7:** Contents of tartrazine (mean and %RSD, *n* = 3) in real samples determined with the proposed methodology and comparison with HPLC reference method.

Sample	[Tartrazine] (mg** **L^−1^)	
	MSPE-UV	HPLC
1	5.5 (1.3)	5.4
2	22.7 (0.8)	22.4
3	5.9 (1.8)	5.7
4	22.8 (2.3)	23.0
5	5.5 (2.6)	5.7
